# Exfoliative esophagitis secondary to tislelizumab: a case report

**DOI:** 10.3389/fonc.2024.1498253

**Published:** 2024-11-29

**Authors:** Mingxing Wang, Qingming Sun, Wanhui Dong

**Affiliations:** Department of Medical Oncology, Lu’an Hospital of Traditional Chinese Medicine Affiliated To Anhui University of Chinese Medicine, Lu’an, China

**Keywords:** tislelizumab, exfoliative esophagitis, immune checkpoint inhibitors, immune-related adverse events, case report

## Abstract

Tislelizumab, as a PD-1 inhibitor, has demonstrated significant efficacy in cancer treatment. However, it may also induce immune-related adverse events (irAEs). This case report describes a patient who developed oral ulcers and dysphagia following treatment with tislelizumab, which was diagnosed as exfoliative esophagitis through endoscopic examination. The condition improved after corticosteroid pulse therapy. A review of the relevant literature revealed no prior reports of immune checkpoint inhibitor (ICI)-related esophagitis cases, prompting an exploration of the potential pathophysiological mechanisms and therapeutic strategies. This report emphasizes the importance of vigilance for rare irAEs during ICI therapy, advocating for early detection and timely intervention.

## 1 Introduction

Esophageal cancer is a significant global health challenge, with substantial variations in incidence and mortality rates across different regions. Globally, it poses a considerable threat to public health. According to the Global Cancer Observatory (GLOBOCAN), which provides critical insights into the burden of cancer worldwide, esophageal cancer was responsible for over 540,000 deaths in 2020, making it the sixth leading cause of cancer-related mortality. Projections indicate that this figure could rise to 880,000 deaths by 2040 due to esophageal cancer (1). The disease is primarily classified into two main types: squamous cell carcinoma (SCC) and adenocarcinoma. This distinction is important, as it influences both treatment strategies and prognosis, with SCC being more prevalent in Asia and Africa, while adenocarcinoma is more common in Western countries (2). As global lifestyles and dietary habits continue to evolve, the incidence of esophageal cancer has been on the rise, highlighting the need for novel therapeutic approaches. The advent of immune checkpoint inhibitors has marked a significant shift in the treatment paradigm for patients with advanced esophageal cancer, offering new hope. Among these immune checkpoint inhibitors, tislelizumab, a monoclonal antibody targeting the programmed cell death protein 1 (PD-1), has shown particular promise and has received approval for the treatment of various malignancies, including esophageal cancer (3). Clinical trials have demonstrated that tislelizumab exhibits promising efficacy and a relatively acceptable safety profile, establishing it as a vital treatment option for individuals with advanced disease. While tislelizumab has demonstrated promising efficacy, its adverse reactions have become a significant concern in clinical practice (4).

Exfoliative esophagitis, although rare, is a severe side effect that can markedly impact patients’ quality of life. While the pathogenesis of exfoliative esophagitis remains incompletely understood, it is known to be closely linked to immune system activation and damage to the esophageal mucosa (5). Currently, the pathogenic mechanisms underlying the development of exfoliative esophagitis induced by tislelizumab are under continuous exploration. Based on existing research and literature, there are multiple potential mechanisms. On the one hand, tislelizumab, as a monoclonal antibody against the PD-1, primarily functions by blocking the interaction between PD-1 and its ligands, PD-L1 and PD-L2. Under normal conditions, the engagement of PD-1 with its ligands inhibits T-cell proliferation and cytokine production, thereby preventing excessive activation of the immune system that could lead to damage to normal tissues. However, when tislelizumab blocks this pathway, the activation state of the immune system is altered, enhancing the activity of T cells, which may mistakenly attack normal tissues such as esophageal mucosa, thereby initiating an inflammatory response in the esophagus, laying the groundwork for the occurrence of exfoliative esophagitis (6). Studies have indicated that following the use of immune checkpoint inhibitors, the expression of some co-stimulatory molecules on the surface of T cells is upregulated, further enhancing the activation of T cells and exacerbating the immune response. On the other hand, cytokines also play a significant role in this pathological process (7). Levels of cytokines such as tumor necrosis factor-alpha (TNF-α) and interferon-gamma (IFN-γ) may change following tislelizumab treatment. TNF-α, which promotes inflammatory responses and apoptosis, can induce esophageal epithelial cells to produce inflammatory mediators and increase vascular permeability, leading to damage and inflammatory cell infiltration of the esophageal mucosa (8). IFN-γ, in turn, enhances the phagocytic function and antigen-presenting capacity of macrophages, prompting the immune system to mount a stronger immune response against esophageal tissues. The abnormal expression and interaction of these cytokines may jointly contribute to the development of exfoliative esophagitis (9). Additionally, literature has pointed out that immune checkpoint inhibitors may affect the balance of the gut microbiota, which is closely related to the immune microenvironment of the esophagus. Disruption of the gut microbiota may indirectly affect the immune status of the esophagus through various pathways, including immune modulation and the production of metabolic byproducts, increasing the risk of developing exfoliative esophagitis (10).

To date, there have been no documented cases in the literature of exfoliative esophagitis induced by tislelizumab or other PD-1/PD-L1 inhibitors. This report describes the first case of severe exfoliative esophagitis occurring in the context of PD-1 inhibition, underscoring the importance of vigilant monitoring and documentation of rare side effects. Therefore, an in-depth investigation of tislelizumab-induced exfoliative esophagitis will enhance awareness among healthcare providers regarding this adverse effect and facilitate the development of more precise management strategies for affected patients.

## 2 Case report

A 63-year-old male patient presented to the Fourth People’s Hospital of Lu’an City on May 3, 2018, with dysphagia. He reported no history of hypertension, heart disease, infectious diseases such as hepatitis and tuberculosis, blood transfusions, or drug allergies. Gastroscopy revealed an elevated lesion with central depression and ulceration in the esophageal segment 26–30 cm from the incisors and a slightly elevated mucosal lesion with superficial erosion at 34–35 cm. Pathological results indicated squamous cell carcinoma of the esophagus (cT3N0M0). Given the patient’s poor physical condition [Karnofsky Performance Status (KPS) = 60], surgery was contraindicated. Instead, he underwent precise radiotherapy for the middle and lower segments of the esophagus and the mediastinal lymph node drainage area from May 21 to July 3, 2018, with a total dose of 60 Gy in 30 fractions. Concurrent oral chemotherapy with capecitabine was administered. Following radiotherapy, the patient’s dysphagia improved. He then received one cycle of chemotherapy with cisplatin 30 mg on days 1–4 and docetaxel 4 mg on day 1, followed by three cycles of single-agent docetaxel. He maintained regular outpatient follow-ups until 2022.

**Figure 1:**
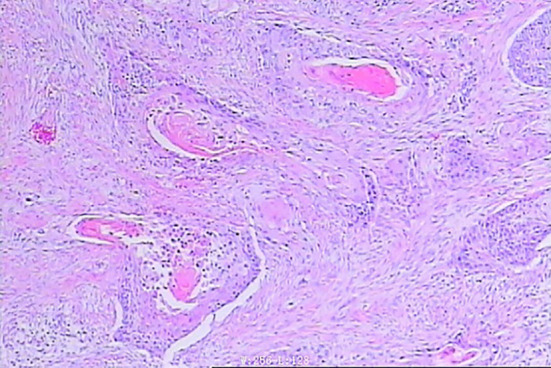
Pathology of esophageal cancer on April 30, 2024.

**Figure 2:**
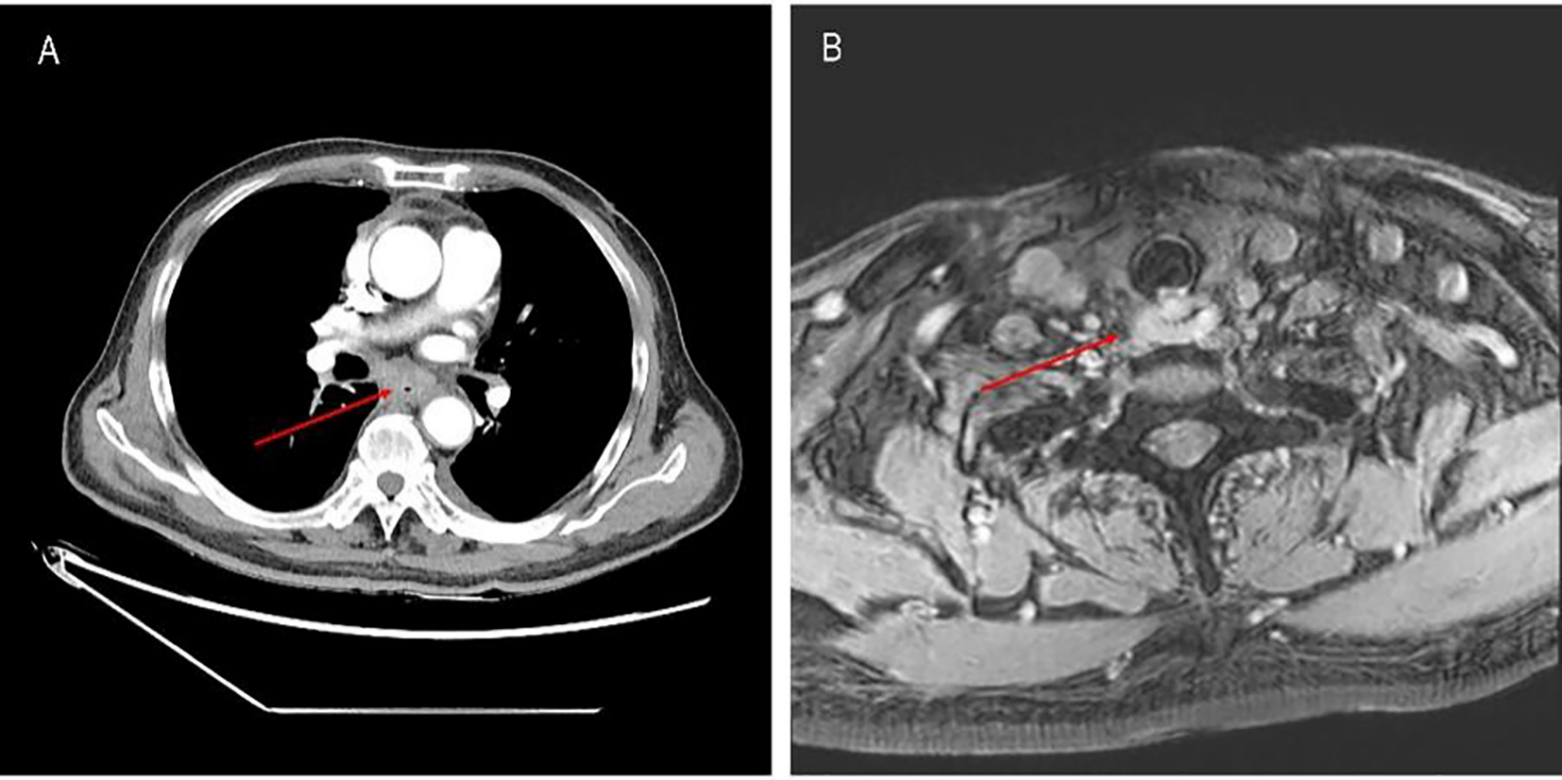
**(A)** Mass in the middle and lower segments of the esophagus before treatment with tislelizumab plus XELOX. **(B)** Mass in the laryngeal area before treatment with tislelizumab plus XELOX.

**Figure 3:**
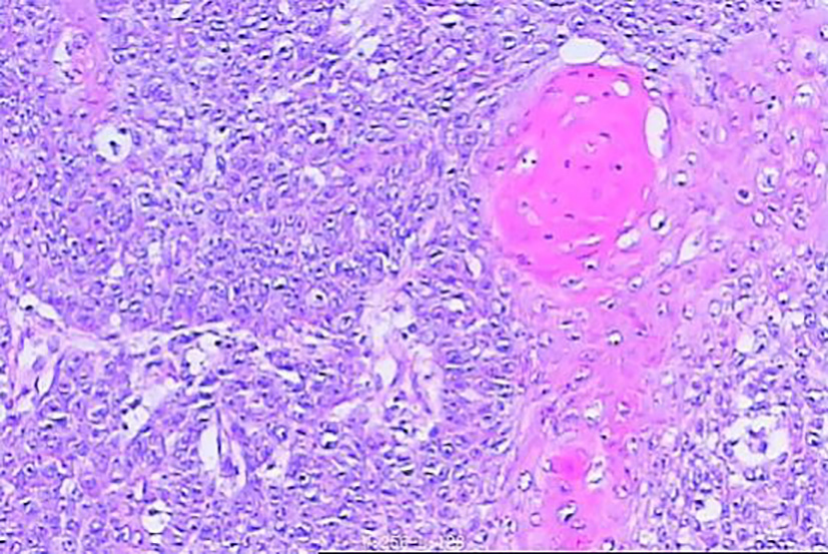
Pathology of laryngeal cancer on May 6, 2024.

**Figure 4:**
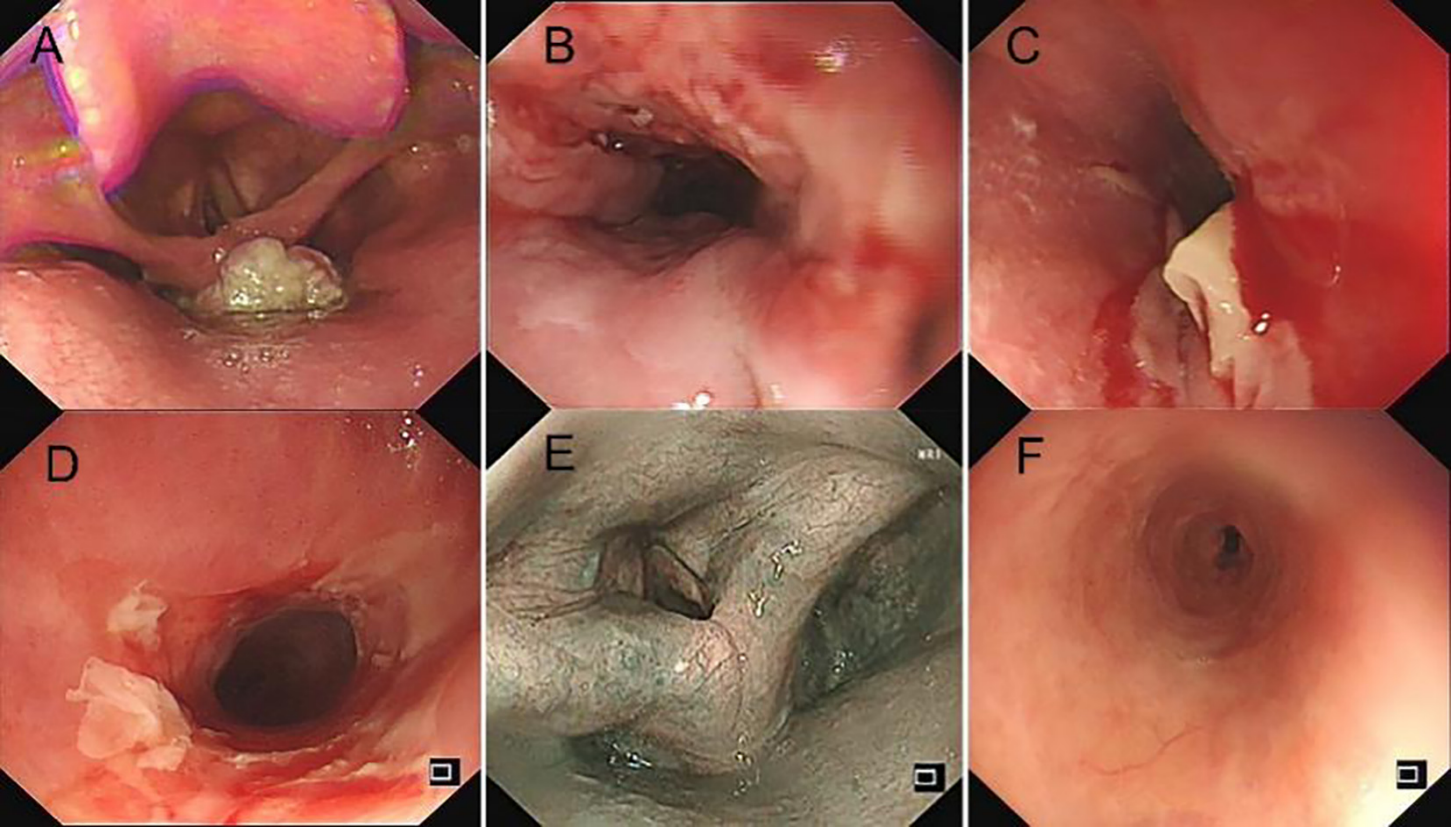
**(A)** Foreign body in the oropharynx before treatment. **(B)** Ulcerative mass at the lower esophagus before treatment. **(C, D)** Exfoliative esophagitis following one cycle of tislelizumab treatment. **(E)** Resolution of oropharyngeal mass after one cycle of tislelizumab and three cycles of raltitrexed treatment. **(F)** Regression of exfoliative esophagitis after 1 month of pulse glucocorticoid therapy.

In April 2024, the patient was admitted to our hospital (Lu’an Hospital of Traditional Chinese Medicine Affiliated to Anhui University of Chinese Medicine) due to choking on rice and vomiting when drinking water. A CT scan on April 29, 2024, suggested thickening of the wall of the middle and lower segments of the esophagus, with air and fluid accumulation in the upper esophagus, indicating a recurrence. On April 30, 2024, an electronic gastroscopy revealed the following: 1) esophageal obstruction (a biopsy was taken later, with the pathology number 202407509) (**Figures 1**, **2**) obstruction in the pharyngeal area. An enhanced CT on April 30, 2024, showed thickening and significant enhancement of the soft tissue behind the hypopharynx, with enlargement of bilateral cervical lymph nodes (right side more prominent). It also revealed thickening and significant enhancement of the wall of the middle and lower segments of the esophagus (**Figure 2A**), with enlargement of lymph nodes under the carina and in the hepatogastric space. An MRI showed an obstruction in the laryngeal area (**Figure 2B**). On May 6, 2024, a biopsy of the laryngeal mass was performed under electronic laryngoscopy, diagnosing laryngeal squamous cell carcinoma, possibly originating from the esophagus (pathology number 202404210) (**Figure 3**). The patient was advised to undergo immunohistochemistry to further clarify the diagnosis but refused. He was then treated with one course of XELOX plus tislelizumab 200 mg on May 9, 2024. On May 28, 2024, the patient developed pharyngeal ulceration and pain. Endoscopy revealed exfoliative esophagitis (**Figure 4**), which was considered likely to be immune-related based on the clinical presentation. He was treated with intravenous methylprednisolone 80 mg pulse therapy and gradually tapered to maintenance therapy with methylprednisolone tablets 4 mg for 1 month, after which he improved. Following chemotherapy with the XELOX regimen on July 19, 2024, and August 14, 2024, the patient’s laryngeal mass has since regressed.

## 3 Discussion

### 3.1 Exfoliative esophagitis: definition, clinical manifestations, and differential diagnosis

Exfoliative esophagitis is an exceedingly rare form of acute esophageal inflammation characterized primarily by dysphagia, chest pain, and potential bleeding. Endoscopically, it presents as the formation of submucosal hematomas on the esophageal surface, with partial or complete separation of the mucosa from the lamina propria, leading to the formation and sloughing of tubular casts. The detection rate accounts for only 0.05% to 0.15% of concurrent gastroscopy examinations, with severe cases capable of causing intense pain and vomiting of large volumes of fresh blood, posing a life-threatening condition. Clinically, the differential diagnosis of exfoliative esophagitis must consider a variety of etiologies, including drug-induced esophagitis (due to mucosal injury from certain orally administered medications), infectious esophagitis (caused by fungal or viral infections), radiation esophagitis, chemical esophageal injury, and systemic diseases (such as pemphigus or myasthenia gravis). Patients with esophageal cancer often experience severe pain associated with their primary lesion and a significant esophageal reaction; if exfoliative esophagitis is not promptly identified, it can greatly increase the risk of extensive secondary infections leading to death (11). Common etiologies of exfoliative esophagitis include dietary factors (ingestion of rough and dry foods or rapid eating), intense vomiting, foreign body impaction, and non-steroidal anti-inflammatory drugs (NSAIDs). A search of databases such as PubMed, Web of Science, and China National Knowledge Infrastructure (CNKI) has revealed that, to date, no literature has reported exfoliative esophagitis induced by tislelizumab or other PD-1/PD-L1 inhibitors. This case serves as the first instance of severe exfoliative esophagitis occurring randomly with the use of a PD-1 inhibitor, highlighting its significance as a cautionary reminder.

### 3.2 Mechanism of action and common adverse reactions of tislelizumab

Tislelizumab is a novel humanized monoclonal antibody against PD-1. It primarily enhances the T cell-mediated immune response against tumor cells by blocking the interaction of the PD-1 with its ligands, PD-L1 and PD-L2 (12). Compared with traditional treatment methods, tislelizumab has demonstrated significant efficacy in the treatment of various tumor types and has become an integral component of cancer immunotherapy. However, with its broader clinical application, associated adverse reactions have been increasingly reported, predominantly immune-related adverse reactions, including rash, thyroid dysfunction (such as thyroiditis), pneumonia, and hepatitis. These reactions are often attributed to the drug’s activation of autoimmune mechanisms. Additionally, patients may exhibit symptoms such as fatigue and diarrhea; the former is typically a result of the enhanced immune response, while the latter is associated with disruptions in intestinal function (13). Furthermore, tislelizumab use may also lead to rare endocrine disorders, such as diabetes or adrenal insufficiency, as well as other serious adverse events, including acute renal failure and anaphylactic shock, which can be fatal in severe cases (14). Although some adverse reactions may be severe, most are mild to moderate and transient and typically respond well to symptomatic treatment, leading to rapid recovery.

### 3.3 Evaluation of drug adverse reaction association

The patient was diagnosed with esophageal squamous cell carcinoma accompanied by laryngeal squamous cell carcinoma upon admission. In accordance with the “Chinese Society of Clinical Oncology (CSCO): Esophageal Cancer Diagnosis and Treatment Guidelines 2024”, the tislelizumab plus XELOX regimen was selected for chemotherapy. Twenty-six days after initiating chemotherapy with this regimen, the patient developed symptoms such as pharyngeal ulceration, pain, and dysphagia, with endoscopic examination suggesting exfoliative esophagitis. Possible predisposing factors for exfoliative esophagitis include the ingestion of fried, hard, and overheated foods, as well as rapid eating; moreover, a normal diet, alcohol consumption, and drug administration may also be contributing factors. Endoscopically, the patient exhibited partial separation of the esophageal mucosa, fulfilling the diagnostic criteria for exfoliative esophagitis. According to the Chinese adverse drug reaction association evaluation, the association between tislelizumab and the occurrence of exfoliative esophagitis in the patient was assessed (**Table 1**): 1) the patient had no prior history of exfoliative esophagitis, did not consume fried or hard foods during hospitalization, and denied a history of alcohol consumption. Thus, dietary influence could be ruled out. Prior to chemotherapy, endoscopy revealed a mass in the lower esophagus without mucosal exfoliation and no abnormal immunological indicators, and the patient had no recent history of surgery or infection. Exfoliative esophagitis occurred subsequent to the administration of tislelizumab in combination with the XELOX regimen, indicating a strong temporal association. 2) The prescribing information for tislelizumab does not list exfoliative esophagitis as a side effect, and there are no relevant reports in the domestic or international literature. However, the blockade of immune checkpoints may lead to immune-related adverse events (irAEs), which include gastrointestinal reactions. The pathophysiology of exfoliative esophagitis involves immune-mediated mucosal damage, where the activation of T cells may lead to the destruction of esophageal mucosal cells (15, 16). 3) After the patient discontinued tislelizumab, the pain gradually subsided, and endoscopy showed a gradual reduction in esophageal mucosal exfoliation, with the esophageal mucosa returning to normal 1 month after combined hormone treatment. 4) The concurrent medications for the patient’s adverse reactions were oxaliplatin and capecitabine. The primary mechanism of action of oxaliplatin is interference with DNA and stimulation of nerves and intestines, leading to peripheral neuropathy, nausea and vomiting, bone marrow suppression, and allergic reactions (17). Capecitabine, through its metabolite fluorouracil, exerts toxicity on epidermal and intestinal cells, thereby causing hand–foot syndrome, nausea and vomiting, bone marrow suppression, and diarrhea (18). Additionally, the patient was administered a dose of capecitabine of 1.5 g each time (twice a day) and oxaliplatin at a dose of 150 mg, all in accordance with CSCO guidelines, and the patient did not develop exfoliative esophagitis after treatment with capecitabine and cisplatin in 2018. Thus, the influence of platinum-based and fluorouracil-based drugs could be excluded. Based on the above analysis, the association rating between tislelizumab and exfoliative esophagitis is “probable”. According to Naranjo’s adverse drug reaction association evaluation principle (19), the score is 6, indicating a strong association (probable correlation) between tislelizumab and exfoliative esophagitis. See **Table 2** for specific scores.

**Table 1:** Evaluation of the association of adverse drug reactions.

No.	Index	Results
1	Is there a reasonable time relationship between drug use and the occurrence of adverse reactions/events?	+
2	Does the reaction conform to the known types of adverse reactions of this drug?	?
3	After drug withdrawal or dose reduction, does the reaction disappear or lessen?	+
4	Does the same reaction event recur when the suspected drug is used again?	?
5	Can the reaction/event be explained by the effects of concomitant drugs, the progress of the patient's condition, and the influence of other treatments?	–

+ means yes, − means no, and ? means unknown.

**Table 2:** Naranjo adverse drug reaction assessment scale.

Related questions	Question scores			Answers	Scores
	Yes	No	Unknown		
1. Are there conclusive reports on tislelizumab-related exfoliative esophagitis?	+1	0	0	No	0
2. Did exfoliative esophagitis occur after the use of tislelizumab?	+2	−1	0	Yes	+2
3. Did exfoliative esophagitis resolve after discontinuation of tislelizumab?	+1	0	0	Yes	+1
4. Did exfoliative esophagitis recur after re-use of tislelizumab?	+2	−1	0	Unknown	0
5. Are there other causes of exfoliative esophagitis?	−1	+2	0	No	+2
6. Does this adverse reaction recur after the application of placebo?	−1	+1	0	Unknown	0
7. Does tislelizumab reach a toxic concentration in the blood or other body fluids?	+1	0	0	Unknown	0
8. Does exfoliative esophagitis worsen with an increase in the dose of tislelizumab or is it relieved with a decrease in the dose?	+1	0	0	Unknown	0
9. Has the patient been exposed to similar drugs and had a similar reaction?	+1	0	0	No	0
10. Is there objective evidence of this adverse reaction with the patient's exfoliative esophagitis?	+1	0	0	Yes	+1
Total score	+6				

A total score of ≥9 indicates a definite association between the drug and the adverse reaction. A total score between 5 and 8 indicates a high probability of an association between the two. A total score of 1–4 indicates that there may be some association. A total score of ≤0 indicates that this association is suspect.

### 3.4 Treatment measures for exfoliative esophagitis secondary to tislelizumab

The pathogenesis of exfoliative esophagitis remains unclear to date. Through the literature review, the treatment measures primarily include the following. 1) Drug treatment. Treatment with TNF-α inhibitors (infliximab) or immunosuppressants (mycophenolate mofetil) is recommended. Infliximab, a TNF-α inhibitor, can specifically inhibit TNF-α, thus playing a therapeutic role in immune-mediated inflammatory diseases (20). Mycophenolate mofetil, as an immunosuppressant, inhibits the proliferation of lymphocytes by synthesizing mycophenolic acid, thereby preventing white blood cells from entering sites of inflammation and graft rejection and further reducing the immune response (21). 2) Improvement of drug administration methods. For drug-induced exfoliative esophagitis, adjustments to the drug administration method can be considered, such as opting for injectable formulations or avoiding orally irritating drugs, and ensuring that patients drink a large amount of water when taking medications to reduce esophageal irritation. 3) Diet adjustment. It is advised that patients consume a soft, mild diet and avoid spicy, acidic, and overheated foods to reduce esophageal irritation. Concurrently, ensuring adequate water intake is essential. 4) Treatment of underlying diseases. Early intervention for underlying diseases that induce exfoliative esophagitis, such as autoimmune diseases, should be undertaken. 5) Hormone pulse therapy. In this case, the patient was administered methylprednisolone 80 mg via intravenous drip for pulse therapy, which was then gradually reduced to methylprednisolone tablets 4 mg for maintenance treatment over 1 month. Additionally, the patient was advised to drink more water and follow a light diet, which led to improvement.

In conclusion, tislelizumab is an effective immunotherapy drug for tumors. Although exfoliative esophagitis caused by it is rare, it requires significant attention from clinicians. A case report indicates that a patient with esophageal cancer developed interstitial pneumonitis after receiving two doses of camrelizumab (each 200 mg), which ultimately led to the patient’s death. This alerts clinicians to remain vigilant when administering immune checkpoint inhibitors at standard doses (22). Clinicians should conduct early detection of gastrointestinal irAEs in patients treated with tislelizumab or other PD-1 inhibitors. For high-risk groups, special attention should be paid to drug administration methods and diet regulation during the diagnosis and treatment process to reduce the risk of exfoliative esophagitis. Furthermore, patients should be regularly followed up after treatment, with monitoring of symptom changes and esophageal mucosa healing, and endoscopic examinations should be performed when necessary to ensure timely identification and treatment of potential complications, thereby improving treatment outcomes and quality of life.

## 4 Limitations and improvements

The limitations were as follows. 1) Limited number of cases. As this is a case report, it involves only one patient, which limits the generalizability and statistical significance of the research findings. 2) Lack of a control group. The study does not include a control group, making it challenging to assess the efficacy and safety of tislelizumab compared to other treatment regimens. 3) Lack of long-term follow-up data. Case reports typically lack long-term follow-up data, making it difficult to evaluate the long-term efficacy and safety of tislelizumab in the treatment of exfoliative esophagitis. Improvement measures were as follows. 1) Increase sample size. Conduct multi-center, large-sample retrospective analyses to increase the number of cases, thereby improving the reliability and generalizability of the research findings. 2) Design controlled studies. In future studies, design randomized controlled trials (RCTs) or cohort studies to provide more robust evidence for evaluating the efficacy of tislelizumab. 3) Long-term follow-up. Implement long-term follow-up studies to assess the long-term efficacy and safety of tislelizumab in the treatment of exfoliative esophagitis. 4) Innovative research directions. Explore the relationship between esophageal micro-ecology and exfoliative esophagitis and investigate how to prevent and manage irAEs by regulating the microbial community, potentially offering new treatment strategies for the management of esophageal irAEs.

## 5 Conclusion

When treating gastrointestinal malignancies with tislelizumab or immune checkpoint inhibitors, it is advisable to use gastric mucosal protective agents early to prevent the occurrence of exfoliative esophagitis. Additionally, early detection of adverse gastrointestinal effects in patients is crucial, with close monitoring of symptoms. Should exfoliative esophagitis occur, timely intervention with glucocorticoids, TNF-α inhibitors, or immunosuppressants is warranted.

## Data Availability

The original contributions presented in the study are included in the article/**Supplementary Material**. Further inquiries can be directed to the corresponding author.
