# Supplementary material for: Exfoliative esophagitis secondary to tislelizumab: a case report
Source: Front Oncol. 2024 Nov 29;14:1498253. doi: 10.3389/fonc.2024.1498253 (PMC11638584; doi:10.3389/fonc.2024.1498253)
Supplement: Supplementary file 1 [file DataSheet1.docx]

14. Y Wang, S Huang, QY Shen, et al. Seventy-nine cases of tislelizumab-related adverse reactions. Chinese Journal of Pharmacovigilance,2024, 21(10): 1148-1153.**Chinese**

**Abstract: Objective** To analyze the regularity and clinical characteristics of adverse drug reactions (ADR) induced by tislelizumab in order to provide references for safe and rational drug use. **Methods** Seventy-nine reports of adverse reactions related to tislelizumab submitted by our hospital to the national ADR Monitoring System from January 1, 2020 to March 31, 2024 were retrospectively analyzed, involving patients’ demographics, types of drugs used, times of adverse reactions, clinical presentations, organs and/or systems involved, severity, management and outcomes, causality assessment, and off-label use. **Results** Among these cases of tislelizumab-related adverse reactions, 88.61% occurred after one to five medication cycles. Affecting multiple organs and systems, the main manifestations included bone marrow suppression, skin toxicity, thyroid dysfunction, and liver function abnormalities. Myasthenic syndrome and anaphylactic shock were serious ADR not specified in drug instructions. After discontinuation of the drug and symptomatic treatment, most of the cases improved. **Conclusion** A few severe adverse reactions of tislelizumab can lead to life-threatening consequences. Close monitoring during clinical use is recommended, with timely ymptomatic treatment to reduce severe adverse reactions.

16. MT Huang, X Song, PL Zhang. A case of pemphigus vulgaris involved esophageal mucosa. Journal of Clinical Dermatology, 2019, 48: 368-70.**Chinese**

**[Abstract]** A case of pemphigus vulgaris involved esophageal mucosa is reported. A 47-year-old female presented with erosion on her oral mucosa with hematemesis for 2 years, and relapsed for 3 days. Dermatological examination showed erosionon the oral cavity palate and gum. Nikolsky sign was positive. Histopathological examination showed hyperkeratosis, acantholysis, intraepidermal blisters with acantholytic cells and red blood cells. Direct immunofluorescence showed reticular deposition of IgG and C3 between the stratified squamous epithelial cells, and negative for both IgA and IgM. Indirect immunofluorescenceshowed positive for anti-desmoglein (Dsg)3 and negative for anti-Dsg1. Gastroscopy revealed exfoliation and bleeding of esophagealmucosa. The diagnosis of pemphigus vulgaris involved esophageal mucosa was made.

1. MM Zhang, ZC Gong, YY Chen. Research progress on oxaliplatin-induced neurotoxicity intraditional Chinese medicine (TCM) and western medical cognition and prevention and treatment by TCM. China Journal of Chinese Materia Medica, 2023, 48: 4610-4619.**Chinese**

**［Abstract］** Chemotherapy is one of the main options in clinical tumor treatment. Although chemotherapy drugs have a good therapeutic effect，they can also cause a series of adverse reactions，such as neurotoxicity. Chemotherapy-induced neurotoxicity is a dose-limiting adverse reaction that significantly affects patients' long-term treatment and quality of life. This article reviewed literature from 2000 to the present on chemotherapy-induced neurotoxicity and found that oxaliplatin was the most frequently used chemotherapy drug. Based on the clinical characteristics of oxaliplatin-induced neurotoxicity, this article summarized the understanding of its pathogenesis from both traditional Chinese medicine (TCM) and western medicine perspectives, discussed the role and mechanism of TCM compounds and monomeric components, and explored the research direction of using cutting-edge biotechnology to reveal the mechanism of oxaliplatin-induced neurotoxicity from a temporal-spatial perspective of intercellular communication and the application prospects of an interdisciplinary model combining TCM pathogenesis, western medicine manifestations, and artificial intelligence in precise intervention decision-making for TCM, aiming to provide research ideas for the prevention and treatment of oxaliplatin-induced neurotoxicity and the development of new drugs.

18. ZP Wang, F Zhang, SH Gao, et al. Research progress of individualized capecitabine administration against colorectal cancer. Chinese Journal of Hospital Pharmacy, 2017, 37: 885-891.**Chinese**

**ABSTRACT:** Colorectal cancer is one of the most common cancers, which has an increased morbidity and mortality and causesthousands of deaths every year. Capecitabine has served for ten years in clinical practice, and it is an important drug againstcolorectal cancer for its superior efficacy and patient compliance, but the drug still has nonnegligible adverse reactions. A correlation between curative effects, toxicities and metabolic enzyme polymorphisms of capecitabine has been discovered by researches, and can be explained by polymorphisms. With the development of the precision medicine, experiments based on molecularbiological research progress, precise and efficient therapeutic regimens are utilized to generate urgent research contents of anticancer agents. Individualized therapeutic regimen can be obtained based on pharmacokinetics and pharmacodynamics, drug interactions and gene polymorphism. The information and relevant clinical application are reviewed in this article.
